# The Spillover Effect of Autonomy Frustration on Human Motivation and Its Electrophysiological Representation

**DOI:** 10.3389/fnhum.2020.00134

**Published:** 2020-04-22

**Authors:** Hui Fang, Xiaoming Wan, Shuyue Zheng, Liang Meng

**Affiliations:** ^1^School of Business Administration, Guangdong University of Finance, Guangzhou, China; ^2^Laboratory of Neuromanagement and Decision Neuroscience, Guangdong University of Technology, Guangzhou, China; ^3^School of Management, Guangdong University of Technology, Guangzhou, China; ^4^School of Business and Management, Shanghai International Studies University, Shanghai, China; ^5^Laboratory of Applied Brain and Cognitive Sciences, Shanghai International Studies University, Shanghai, China; ^6^Wharton Neuroscience Initiative, The Wharton School, University of Pennsylvania, Philadelphia, PA, United States

**Keywords:** self-determination theory, autonomy frustration, controlling context, autonomous motivation, event-related potentials, reward positivity (RewP)

## Abstract

It is a commonplace that some people may adopt a controlling style, which brings about autonomy frustration to others. Existing studies on autonomy frustration mainly examined its effect in the primary thwarting context, ignoring its potential spillover to subsequent activities. In this study, we examined whether prior autonomy frustration would have a sustaining negative impact on one’s motivation in another autonomy-supportive activity that follows. In this electrophysiological study, participants worked on two irrelevant tasks organized by two different experimenters. We adopted a between-group design and manipulated participants’ autonomy frustration by providing varied audio instructions during Session 1. In Session 2, all participants were instructed to complete a moderately difficult task that is autonomy-supportive instead, and we observed a less pronounced reward positivity (RewP) difference wave and a smaller P300 in the autonomy-frustration group compared with the control group. These findings suggested that the negative influence of autonomy frustration is longstanding and that it can undermine one’s motivation and attention in a following activity that is autonomy-supportive itself. Thus, our findings provided original neutral evidence for the adverse intertemporal effect of autonomy frustration, and suggested important practical implications.

## Introduction

Intrinsic motivation is typically defined as the performing of an activity out of interest, curiousness and enjoyment (Deci and Ryan, 1985b). As a broader construct that encompasses intrinsic motivation, autonomous motivation describes one’s willingness and volition based on either values or interests (Hagger and Chatzisarantis, 2016; Kuvaas et al., 2016; Meng and Ouyang, 2020). Previous studies have consistently suggested one’s autonomous motivation level to be a crucial factor in affecting job performance, innovative behaviors, work engagement as well as academic attainment (Guo et al., 2014; Nunez and Leon, 2016). After all, as indicated by self-determination theory, interest in and identification with an activity are much more powerful and persistent motivators compared with other controlled forms motivation (Ryan and Deci, 2017). Given the significance of autonomous motivation, researchers endeavor to identify its sources, and the elements which would either facilitate or impede one’s autonomous motivation. For instance, it was found that when individuals were given choices in an activity, they perceived themselves to be autonomy-supported to a greater extent and then had greater autonomous motivation (How et al., 2013; Meng and Ma, 2015). Conversely, the controlling context which would frustrate one’s perceived autonomy was reported to erode autonomous motivation (Reeve, 2009).

When examining the situational and contextual factors of autonomous motivation, self-determination theory provides the most comprehensive theoretical framework, as well as the strongest explanatory and predictive power (Deci and Ryan, 2000). As one of the fundamental psychological needs, autonomy describes one’s propensity toward a sense of agency and self-determination (Ryan and Deci, 2017), the satisfaction of which brings about enhanced autonomous motivation, optimal functioning and greater happiness (Van Den Broeck et al., 2016). When subjective initiative got oppressed and an individual was pressured to perform certain actions and behaviors in a required manner, one got to experience autonomy frustration. Among the adverse influencers of autonomy, a controlling context would result in autonomy frustration, which is further accompanied by a series of negative outcomes, from decreased autonomous motivation, lower task engagement to poor task performance (Reeve, 2009; Earl et al., 2017). Elements that contribute to a controlling context include orders and directives, the setting of deadlines, surveillance, and the association between the performing of an activity with monetary rewards, all of which have been sufficiently established in existing literatures (Reeve and Jang, 2006; Ryan and Deci, 2017).

In our daily life, the controlling managerial style is not uncommon. For example, many managers frequently employ rewards, orders and directives and set deadlines to motivate/manage their employees and improve job performances. However, the above-mentioned administrative measures would frustrate one’s autonomy, and bring about job burnout and counter-productive behavior (Van Den Broeck et al., 2014; Gillet et al., 2015b). In the educational setting, many teachers are inclined to adopt a controlling instructional style, as they would dismiss students’ perspectives and opinions, and compel them to think or behave in certain manners (Assor et al., 2005). Such behaviors were found to thwart students’ perceived autonomy, which predicted classroom disengagement and bullying behaviors among students (Hein et al., 2015; Jang et al., 2016). While the dark side of the controlling managerial/instructional style has been sufficiently revealed, many managers/teachers still adopted such a style. This inclination is the result of their perceived pressures imposed from either superordinate (demands from administrators) or subordinate sources (employees’ and students’ demotivation in an activity) (Reeve, 2009). In fact, the negative influence of adopting a controlling style can be even more far-reaching, which may spill over afterward. While this prediction has not been adequately examined so far, a pioneering study proposed a *trans*-contextual model of autonomous motivation in the educational setting, which suggested that autonomy satisfaction may have a spillover effect. According to this model, students’ autonomous motivation toward physical activities at school was associated with that outside of school (Hagger and Chatzisarantis, 2016). In this study we predict to observe a similar spillover effect for autonomy frustration.

This study aims to broaden the existing understanding of autonomy frustration and to assess its potential spillover effect. Given that previous studies have consistently suggested autonomy frustration to be associated with amotivation, disengagement, and undermined autonomous motivation in a given task (Hofferber et al., 2016; Earl et al., 2017), we further propose that prior autonomy frustration may cause one’s autonomous motivation to recede in a following irrelevant activity. Our proposition can find indirect support from several sources of literatures. To begin with, previous longitudinal studies suggested that students grown up in controlling families which thwarted their autonomy were more likely to develop depressive symptoms (Shahar and Henrich, 2013), commit oppositional defiance (Vansteenkiste et al., 2013), behave more aggressively (Joussemet et al., 2008), and have lower happiness (Assor et al., 2004). Moreover, our proposition is in accord with the observation that, compared with their peers, law school students educated in a controlling context had a lower autonomous motivation in their first jobs after graduation (Sheldon and Krieger, 2006). In addition, individuals who experienced relatedness frustration (in forms of ostracism or rejection) beforehand were found to display more aggressive behaviors and become less altruistic toward others in following stages (Twenge et al., 2001, 2007). Taken together, we consider that prior autonomy frustration resulting from the controlling context may have far-reaching negative consequences, and propose that prior autonomy frustration may spill over to an irrelevant activity. To be specific, we predict that autonomy-frustrated individuals may still have undermined autonomous motivation in an irrelevant activity that is autonomy-supportive instead.

To test this prediction, we adopted a between-subject experimental design. Upon arrival at the laboratory, participants were randomly designated to either the control group or the autonomy-frustration group. In the first session, all participants were instructed to play the Tangram puzzle under the audio instructions played by the experimenter. We prepared controlling instructions for the experimental group to induce autonomy frustration, while provided neutral ones for the control group. Then, participants took part in a moderately difficult stopwatch task organized by a different experimenter. To satisfy one’ autonomy, in this session they received several choices. To learn about the influence of previous autonomy frustration on one’s autonomous motivation in the following activity, we compared one’ autonomous motivation and attention level in the stopwatch task between the two groups. Considering that autonomous motivation and attention are implicit and difficult to be measured by traditional methods, we resorted to electroencephalography for a more reliable measurement.

The reward positivity (RewP) is a positive-going event-related potential component, which generally reaches its maximum magnitude around 250–300 ms upon feedback onset, which is most pronounced at frontal-central electrodes (San Martin, 2012). Previous studies consistently demonstrated that it is more pronounced for the positive feedback compared with the negative one. Drawing upon the motivational significance theory of RewP, its amplitude is responsive to the outcomes’ motivational and/or affective significance. To rule out the potential influence of individual differences in the baseline RewP magnitude, most studies examining RewP employed a difference wave approach. Thus, the RewP difference wave (the mean amplitude in response to wins minus that to failures) represents a rapid subjective evaluation of the motivational significance (Gehring and Willoughby, 2002; Yeung et al., 2005). Support for this can be found in previous studies reporting that a more pronounced RewP difference wave corresponds with greater perceived motivational significance (Masaki et al., 2006; Ma et al., 2011; Meng and Ma, 2015; Wei et al., 2020). For example, when the participants were instructed to perform a series of calculation tasks that require different amount of cognitive effort, a larger RewP difference wave would be observed when the participants were evaluating the outcome feedback for multiplications compared with additions. While the participants were always provided with the same amount of monetary rewards once whey won, the more effort-requiring task being completed enhanced the motivational significance of receiving the performance feedback (Ma et al., 2014). Following these pioneering literatures, we applied the amplitude of RewP difference wave to measure one’ motivation level in Session 2. As we theorized that prior autonomy frustration would spill over to the subsequent irrelevant activity, we predicted to observe a less pronounced RewP difference wave in the autonomy-frustration (experimental) group in comparison with the control group.

P300 is a positive-going deflection, the amplitude of which maximizes at approximately 300–600 ms after feedback onset. The most representative P300 waveform is observed at frontal and parietal recording sites and is maximal at central-parietal regions (Nieuwenhuis et al., 2005; Polezzi et al., 2009). Previous studies consistently reported that the magnitude of P300 reflects one’s attention resource allocation (Donchin and Coles, 1988; Wu et al., 2011). For instance, a line of studies have shown that stimuli that appeared less frequently would elicit a larger P300 than more frequently occurring ones (Johnson and Donchin, 1980; Wu et al., 2011). As motivation is one of the important influencing factors of one’s attention level, the magnitude of P300 was found to reflect the motivational significance of the feedback (Yeung et al., 2005; San Martin, 2012). Support for this assertion can be found in studies that adopted the gambling task. When receiving self-relevant feedback (e.g., feedback for the participant) in contrast to self-irrelevant one (e.g., feedback for one’s counterpart), the feedback became more motivationally significant. As the participants would pay greater attention to the feedback in the former case, a more pronounced P300 was observed (Fukushima and Hiraki, 2009; Leng and Zhou, 2009). In line with our prediction on RewP, we hypothesized that a less pronounced P300 would be observed in the autonomy-frustration group in comparison with the control group.

Besides examining the intertemporal influence of prior autonomy frustration on one’ autonomous motivation in a different autonomy-supportive activity, we would like to explore the role of causality orientations in such a process. According to self-determination theory, when performing the same behavior, individuals may vary in their interpretations due to dispositional differences. Some may interpret it as self-directed, while others incline to see it as controlled by external factors. There are three types of dispositional tendencies, or causality orientations, which are autonomy, controlled and impersonal, respectively (Deci and Ryan, 1985a; Ryan and Deci, 2017). The two causality orientations most relevant to the current study are autonomy and controlled orientations. Individuals with autonomy orientation try to follow their hearts and perform self-determined behaviors. They are more likely to deem external elements such as rewards and instructions as instructive and supportive. They also tend to display higher intrinsic/autonomous motivation in any activity (Hagger and Chatzisarantis, 2011). In contrast, control-oriented individuals incline to be motivated by external factors like rewards and deadlines, and are more likely to exhibit maladaptive outcomes (Knee and Zuckerman, 1998) and have lower intrinsic/autonomous motivation (Deci and Ryan, 1985a). Furthermore, empirical evidence indicated that control-oriented individuals are more likely to suffer the destructive effect that external rewards have on their intrinsic motivation (Hagger and Chatzisarantis, 2011). Following these pioneering literatures, in this study we propose that, after suffering autonomy frustration control-oriented participants would experience the destructive spillover effect of autonomy frustration to a greater extent and have an even lower autonomous motivation (measured by the amplitude of RewP difference wave) in a subsequent autonomy-supportive activity compared with autonomy-oriented participants.

## Materials and Methods

### Participants

The study was approved by the local institutional review board. Before we started this experiment, we conducted a power analysis with a medium effect size (*F* = 0.4) and an error probability (*a*) of 0.05 to figure out the proper sample size. Accordingly, 46 university students who were registered in a large comprehensive university located in southern China volunteered to take part in the experiment in exchange for 40 RMB (about $6 USD) as compensation. The participants ranged in age from 19 to 23 years (*M* = 20.13, *SD* = 0.91), among which 50% were female. All participants were right-handed and had either normal or corrected-to-normal vision, none of which stated a history of neurological disorders or mental diseases. All participants were required to give written informed consent in accordance with the Declaration of Helsinki. They were randomly allocated to the experimental group and the control group. Two participants with excessive recording artifacts were excluded, leaving 44 participants (22 males; a total of 21 participants in the control group) for the final analysis.

### Experimental Paradigms, Design and Tasks

**FIGURE 1:**
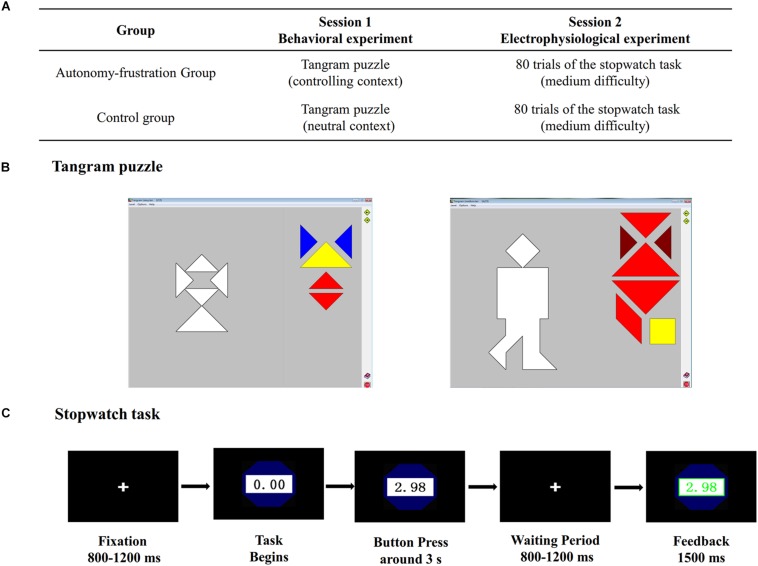
Demonstration of the experimental paradigm. **(A)** The experimental procedure; **(B)** Tangram puzzle interface; **(C)** The stopwatch task procedure.

During the participant recruiting stage, we emphasized that two experimenters organizing two irrelevant experiments decide to recruit participants together as durations of both experiments are rather short. Thus, once a participant candidate signs up, he/she has to first take part in a behavioral experiment and then an electrophysiological experiment (see Figure 1A). The two experiments were conducted in different rooms and were organized by different experimenters, reinforcing the idea that they were irrelevant. Upon the participants’ arrival at the lab, they were again informed that their agreement to join in the experiment means participating two separate experiments which were chunked together only for recruitment convenience purpose. Then, the first experimenter guided the participant into a room prepared for behavioral experiments to work on the first task, which was a computerized Tangram puzzle. Participants in both groups were instructed to construct preset patterns using the geometrical forms provided to them (see Figure 1B). The operation methods and rules were carefully introduced.

While the experimental tasks as well as their sequence were exactly the same for participants in both groups, they were provided varied instructions. Experimental group participants were informed to comply with the audio directives throughout the game. The instructions were well prepared to construct a controlling context. In previous studies, this approach has been widely applied to induce autonomy frustration (Reeve et al., 1999; Radel et al., 2013). Specifically, the instructions comprised of frequent deadlines to accomplish the figures (e.g., “You only have 2 min to complete the figure.”), incorporated orders and requests (e.g., “You started the game 1 min ago, now please don’t deal with figure 8 anymore. Go straightly to figure 9.”), and contained disclosure of solutions (e.g., “Do not forget to place all shape elements.”). In addition, participants were informed that the experimenter would monitor them through the one-way mirror installed in the back wall, and that they should strictly follow the audio instructions during the game.

On the contrary, participants in the control group were informed that the experimenter would play instructions by audio to help them play the game. Consistent with the experimental group, the audio guidance involved the same temporal directions to avoid influencing participants’ competence in any expected manner. However, these instructions did not contain any imperative component. Sample instructions include “When you finish the first figure, it will automatically jump to the next figure.” and “This game normally requires 3 min.” The same speaker’s voice was applied for both groups. After the first experiment terminated, the same experimenter came back and thanked the participant. Following that, participants were invited to accomplish a post-experiment questionnaire about their perceived autonomy frustration in the first task. Then, the experimenter guided them to another room for electrophysical experiments, where another experimenter took charge.

**FIGURE 2:**
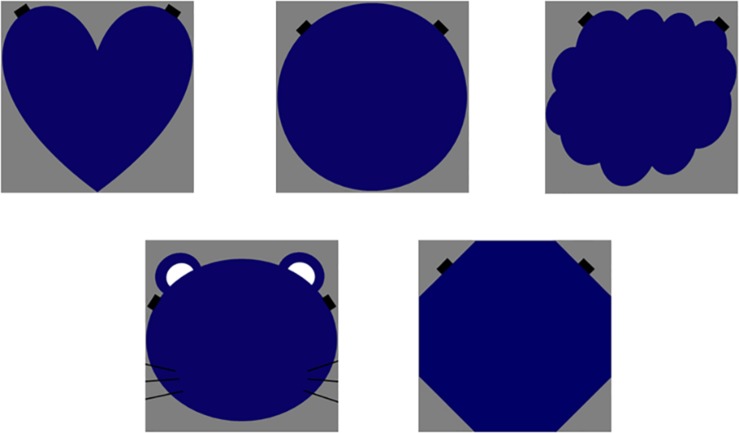
Five stopwatch pictures provided in the stopwatch task. They only vary in their appearance, while difficulties of the task are identical.

In the second electrophysiological experiment, all participants were guided to sit in a dimly lit, electrically shielded and sound-attenuated experimental cubicle. The experimenter introduced the stopwatch task to all participants and instructed them to use a keypad to respond. As illustrated in Figure 1C, the participants were instructed to stop a running watch around 3 s with the right thumb. The whole experiment included 80 trials. No specific instructions were played by audio, so they can play the game at their own pace throughout the experiment. To provide a chance to restore one’s sense of autonomy, participants can voluntarily choose the task icon they like before entering the stopwatch game. We carefully designed five task icons in advance (see Figure 2), including a heart-shape icon, a leaf-shape icon and so on. Moreover, similar with a pioneering study examining autonomy support (Meng and Ma, 2015), we provided two versions of stopwatch games, the discrepancy of which lies in the pre-defined success time window (2.85–3 s for Task A, and 3–3.15 s for Task B). After each 20 trials, participants have an opportunity to choose the game version they like. It is worth noting that participants would receive a fixed amount of money irrelevant to their task performances. As the second task was conducted in a non-controlling context, we believe there were not any controlled sources of motivation, which makes it possible for us to compare the autonomous motivation in the stopwatch task between the two groups.

As depicted in Figure 1C, within each trial, a cross symbol was firstly presented for 800–1200 ms, then a stopwatch would automatically start. If the response time fell into the specified interval, task performances (the exact stop time) would be shown in green. If not, in red. The inter-trial interval lasted for 600–1000 ms. A series of pilot studies were implemented to determine the time windows (i.e., difficulty) of the stopwatch task so that participants could succeed on approximately 50% of the trials. Before the second experiment formally started, all participants were allowed to have a 10-trial practice to familiarize with the stopwatch task. Stimuli, recorded triggers and behavioral responses were presented by E-Prime 2.0 (Psychology Software Tools, Pittsburgh, PA, United States). Following the second experiment, participants were firstly instructed to complete a scale on their perceived competence satisfaction in the stopwatch game, and were then debriefed and paid accordingly.

### Measurements

#### Causality Orientations

We applied the General Causality Orientations Scale to measure one’s causality orientations during the initial screening stage (Deci and Ryan, 1985a). The scale includes 12 written vignettes and participants were faced with three alternatives in each vignette, one concerning an autonomy-oriented item, another control-oriented, and a final one impersonally oriented. Participants were instructed to evaluate their likelihood to respond in each manner on a 7-point Likert scale (1 = “Totally disagree”; 7 = “Totally agree”). We mainly assessed participants’ autonomy orientation and control orientation in this study. The Cronbach’s α are 0.62 and 0.76, respectively.

#### Autonomy Frustration and Competence Satisfaction

We assessed participants’ extent of autonomy frustration in the Tangram puzzle and competence satisfaction in the stopwatch task using two 4-item subscales adapted from the “Work Domain Basic Psychological Need Satisfaction and Frustration Scale” originally developed by Chen et al. (2015) and Schultz et al. (2015). A sample item for autonomy frustration is “When I was playing the Tangram puzzle, I perceived that I have to follow instructions.” A sample item for competence satisfaction is “I felt confident that I can do well in the stopwatch task.” The Cronbach’s α of the autonomy frustration subscale and the competence frustration subscale are 0.758 and 0.920, respectively. Participants were instructed to respond to all items on a 7-point Likert scale (1 = “Totally disagree”; 7 = “Totally agree”).

#### Electroencephalogram Data Recordings and Analyses

Electroencephalogram data were recorded by the eego amplifier, applying a Waveguard Electroencephalogram Cap with 64 Ag/AgCl electrodes (both made by ANT Neuro, Enschede, Netherlands). The channel data went through online band-pass-filter from 0.1 to 100 Hz and were recorded at a sampling rate of 500 Hz. When all electrode impedances were reduced to less than 10 kΩ, we began the stopwatch experiment. We used the left mastoid as online reference. In off-line re-reference, we employed the averaged left and right mastoids.

During off-line data analyses, electroencephalogram data went through standard data processing procedures conducted by ASALab 4.10.1 (ANT Neuro, Enschede, Netherlands). The pre-processing steps are as follows: (a) a digital low-pass filter at 30 Hz (24 dB/octave); (b) identification and collection of ocular artifacts using the algorithm embedded in the ASALab program; (c) segmentation of −200/+800 ms around feedback stimuli onset; (d) baseline correction (the waveform from −200 ms to the feedback stimuli onset served as baseline); (e) artifact detection (trials containing amplifier clippings, bursts of electromyography activity, or peak-to-peak deflections that exceeded ±100 μV were all deleted from the final within-subject averaging).

In this study we focused on the RewP as well as P300, which were consistently demonstrated to be related with feedback processing and outcome evaluation in previous event-related potential studies (Proudfit, 2015; Muhlberger et al., 2017). As amplitude of RewP win-lose difference wave generally reaches its peak at frontocentral electrodes around 300 ms upon feedback onset (Ullsperger et al., 2014; Oemisch et al., 2017; Fernandes et al., 2018), we analyzed its mean voltage within 220–320 ms upon feedback presentation over Fz and FCz channels. Moreover, in line with existing literatures, we calculated the mean voltage of the P300 within 320–470 ms over CZ and CPZ channels (Ferrari et al., 2008; Polezzi et al., 2009). As electrode is not suggested to be included as an additional factor during event-related potential data analyses (Luck and Gaspelin, 2017), we applied electrode clusters for the RewP (F1, FZ, F2, FC1, FCZ, and FC2) and P300 (C1, CZ, C2, CP1, CPZ, and CP2) analyses in this study.

## Results

### Manipulation Check

Results from an independent sample *t*-test indicated that participants in the experimental group (*M* = 4.92, *SD* = 0.63) experienced a higher level of autonomy frustration compared with control group participants [*M* = 2.89, *SD* = 1.07, *t*(42) = −7.74, *p* < 0.001, Cohen’s *d* = 2.31] when working on the Tangram puzzle. Meanwhile, one’s autonomy orientation [*t*(42) = 0.23, *p* = 0.82, Cohen’s *d* = −0.08] and control orientation [*t*(42) = 0.44, *p* = 0.66, Cohen’s *d* = −0.13] were not significantly different between the two groups. Besides, there was no significant between-group difference in one’s perceived competence satisfaction [*t*(42) = 0.83, *p* = 0.41, Cohen’s *d* = −0.25] in the stopwatch task. These findings indicated that our manipulations were successful.

### Task Performances

According to the results of independent sample *t*-tests, there were no significant between-group differences in success rate [*M*_*experimental*_ = 0.49 (*SD* = 0.12), *M*_*control*_ = 0.51 (*SD* = 0.15); *t*(42) = −0.44, *p* = 0.67, Cohen’s *d* = −0.15] or the mean error [*M*_*experimental*_ = 0.07 (*SD* = 0.01), *M*_*control*_ = 0.07 (*SD* = 0.01); *t*(42) = −0.38, *p* = 0.71, Cohen’s *d* = −0.13] in the stopwatch task. In our study, the mean error is defined as the absolute value of discrepancy between the responding time and the target one (3 s).

### Event-Related Potential Results

#### Reward Positivity (RewP)

**FIGURE 3:**
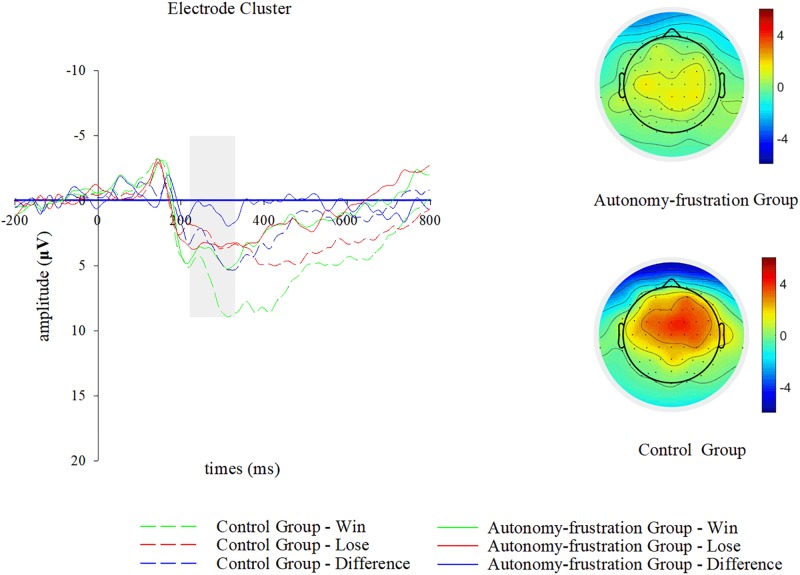
Reward positivity (RewP) results of the chosen electrode cluster (F1, FZ, F2, FC1, FCZ, and FC2) during outcome evaluation. Grand-averaged event-related potentials waveforms of RewP and its win-lose difference wave are shown for group (autonomy-frustration group vs. control group) and outcome (win vs. lose) conditions. Scalp topographic distributions of the difference wave are plotted for both groups, and the bar ranges from –4 to 4 μV.

Mean voltage amplitudes of the RewP (see Figure 3) were 3.71 μV (experimental group-win), 3.43 μV (experimental group-lose), 5.73 μV (control group-win), and 3.02 μV (control group-lose) in respective experimental conditions. ANOVA results of the RewP revealed a main effect of outcome [*F*(1,42) = 23.91, *p* < 0.001, η^2^ = 0.36], and participants presented a more positive RewP in the success condition (*M* = 4.72, *SD* = 0.47) compared with the failure condition (*M* = 3.22, *SD* = 0.48). Meanwhile, the main effect of group was not significant [*F*(1,42) = 0.82, *p* = 0.37, η^2^ = 0.02]. We observed a significant interaction effect (see Figure 3) for outcome × group [*F*(1,42) = 15.74, *p* < 0.001, η^2^ = 0.27], which suggested that amplitude of the RewP difference wave was less pronounced in the experimental group (*M* = 0.28, *SD* = 0.42) than in the control group (*M* = 2.73, *SD* = 0.44). Subsequent simple effect analyses showed that positive feedback evoked a more positive deflection than the negative one only in the control group [*F*(1,20) = 30.82, *p* < 0.001, η^2^ = 0.61], but not in the experimental group [*F*(1,22) = 0.56, *p* = 0.46, η^2^ = 0.03]. In addition, there was a significant between-group difference in the wining condition [*F*(1,42) = 4.69, *p* = 0.04, η^2^ = 0.10], but not in the losing condition [*F*(1,42) = 0.18, *p* = 0.68, η^2^ = 0.01].

**FIGURE 4:**
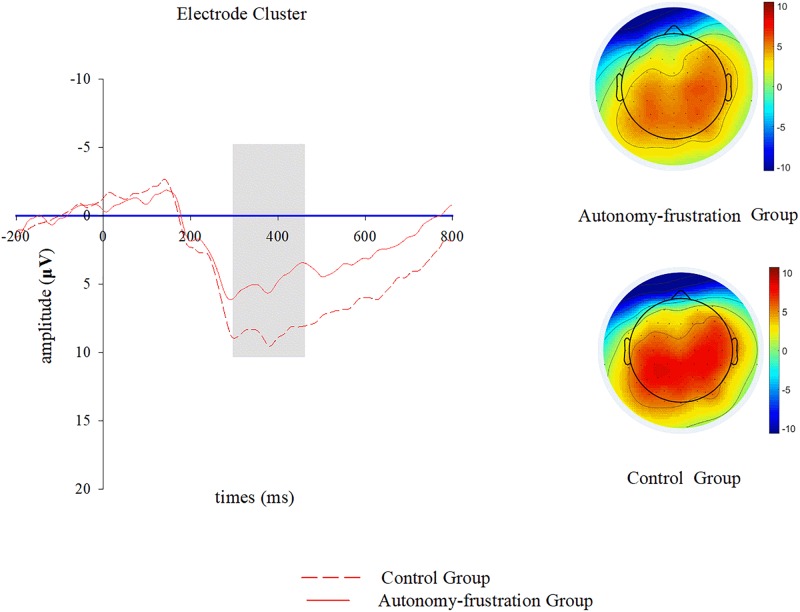
P300 results of the chosen electrode cluster (C1, CZ, C2, CP1, CPZ, and CP2) during outcome evaluation. Grand-averaged P300 are shown for both groups (autonomy-frustration group vs. control group). Scalp topographic distributions of the P300 are plotted for both groups, and the bar ranges between −10 and 10 μV.

Incorporating electrophysiological and self-report data, we observed that the level of autonomy frustration in the Tangram puzzle is negatively correlated with the mean amplitude of RewP win-lose difference wave (*r* = −0.52, *p* < 0.001). Moreover, correlational results suggested that control orientation is negatively correlated with the mean amplitude of RewP difference wave (*r* = −0.56, *p* = 0.006) in the experimental group, and such a relationship was not observed in control group participants (*r* = −0.12, *p* = 0.59). Moreover, we did not observe significant correlations between one’s autonomy orientation and the mean amplitude of RewP difference wave in either the experimental group (*r* = 0.19, *p* = 0.39) or the control group (*r* = 0.34, *p* = 0.12).

#### P300

ANOVA analysis of mean voltage amplitudes between 320 and 470 ms following feedback onset showed a main effect of group [*M*_*E*_ = 3.45, *M*_*C*_ = 5.47, *F*(1,42) = 5.22, *p* = 0.03, η^2^ = 0.12]. Combining electrophysiological and self-report data, we observed that the rated autonomy frustration in the Tangram puzzle negatively correlated with the mean P300 amplitude (*r* = −0.34, *p* = 0.03), while there were no significant correlations between the mean P300 amplitude and one’s autonomy orientation (*r* = −0.01, *p* = 0.96) or control orientation (*r* = −0.01, *p* = 0.98).

## Discussion

Autonomy is proposed as a fundamental psychological need essential for human beings’ optimal functioning and psychological well-being (Ryan et al., 2006). A considerable body of studies have shown that one’s perceived autonomy frustration in an activity predicts a series of negative outcomes, such as job burnout, turnover intention, ill-being, as well as counterproductive behavior (Bartholomew et al., 2014; Van Den Broeck et al., 2014; Gillet et al., 2015a). However, whether the negative impact of autonomy frustration would spread to another activity is elusive and awaits investigation. The current study aims to examine whether experienced autonomy frustration would spill over to a subsequent irrelevant task even if it provides the opportunity for autonomy restoration.

Social environments encompassing deadlines, surveillance, orders and directives have been shown to be controlling (Amabile et al., 1976; Reeve and Jang, 2006; Radel et al., 2014) and then bring about autonomy frustration (Pelletier et al., 2001; Gagne and Deci, 2005; Hein et al., 2015). Thus, we played audio instructions incorporating the controlling elements to induce autonomy frustration during Session 1. This approach is in accordance with a few pioneering studies (Reeve et al., 1999; Radel et al., 2014; Weinstein et al., 2017). Specifically, when participants in the experimental group were working on the first task, we played controlling audio instructions incorporating frequent deadlines, specified solutions, orders and requests. Meanwhile, neutral audio instructions which did not contain any imperative components were played for participants in the control group. We found a smaller RewP difference wave and P300 in the autonomy frustration group than in the control group. Besides, for individuals who experienced autonomy frustration, their control orientation negatively correlated with amplitude of the RewP difference wave. Based on cognitive implications of RewP and P300, our findings suggest that prior autonomy frustration would have a long-term negative influence, as it reduced one’s motivation and attention levels in a subsequent activity out of the primary thwarting context. In addition, one’s control orientation was found to aggravate this negative impact.

At first glance, findings of this study seem to be in conflict with a line of studies conducted by Radel et al. (2013, 2014). As autonomy is a fundamental psychological need, when faced with autonomy frustration, it is unlikely that individuals would just sit there and do nothing. Indeed, according to the theoretical prediction of self-determination theory, when faced with need frustration, individuals may take actions to restore the thwarted need via self-regulation, and the restorative process may even be subconscious (Fiske, 2004; Veltkamp et al., 2009). In a pioneering behavioral experiment that has a similar design with ours, Radel and colleagues found that individuals who experienced autonomy frustration showed stronger intrinsic motivation in a subsequent easy and autonomy-supportive task. It is worth noting that the second task was a computerized Sokoban puzzle, which is fairly easy (Radel et al., 2014). On the contrary, the second task in our study was set to be moderately difficult, the success rate of which was approximately 50%. Our setting of task difficulty was in accordance with the requirement and guideline of RewP data analysis. To properly compare the RewP amplitude between different conditions, researchers have to make sure that the number of winning and losing trials are balanced, and that the success rates are around 50% (Holroyd and Coles, 2002; San Martin, 2012). Otherwise, the magnitude of RewP between different conditions will no longer be comparable.

We consider that differences in the difficulty of the second task may explain differences in the observed results. In the study conducted by Radel and colleagues, an autonomy frustration-induced compensation effect was observed, as participants showed greater interest in another easy task which could help restore their autonomy. In our study, while the second task was autonomy-supportive, its difficulty was not low enough, and autonomy-frustrated participants reported only medium level of competence satisfaction (*M* = 4.21, *SD* = 1.08). When this was the case, participants’ autonomy restorative process might be inhibited, which explains the spillover effect observed in our study. In fact, Radel et al. (2013) suggested that one’s perceived competence might play an important role in the autonomy restoration process. Our finding is also consistent with a recent literature indicating that there are prerequisites and boundary conditions for the restoration of competence, another fundamental psychological need (Fang et al., 2019). Taken together, these findings indicate that the restorative process of autonomy may not always be activated. Thus, other boundary conditions for such a process deserve further investigation.

Findings of this study extend the scope of need frustration and restoration research. Up to now, research on need frustration predominately focused on its immediate effect, in other words, the influence of need frustration in its original thwarting context. Following a few pioneering studies (Radel et al., 2013, 2014; Fang et al., 2017, 2018), in this study we examined the intertemporal effect of autonomy frustration outside of its primary thwarting context. Consistent with several previous studies (Sheldon and Krieger, 2006; Ryan and Deci, 2017), we further confirmed that the negative effect of autonomy frustration can be far-reaching and may spread to subsequent irrelevant tasks. In addition, one of our major contributions is the finding that control causality orientation would aggravate the spillover effect of autonomy frustration. Based on the results of correlation analyses, one’s control orientation is negatively correlated with the motivation (measured by the amplitude of the difference wave of RewP) in the second task, a phenomenon only observed in the experimental group. Hence, among participants whose autonomy got thwarted, control-oriented ones suffered more from prior autonomy frustration. It seems that they declined to restore the undermined autonomy in the second autonomy-supportive task and showed less autonomous motivation in the stopwatch game compared with those with a predominate autonomy causality orientation. This finding showed that individuals with control causality orientation are more likely to suffer the destructive influence that prior autonomy frustration exerted on one’s motivation in the subsequent task, which is in line with findings that control-oriented individuals are more inclined to suffer the detrimental influence that external factors exerted on one’s intrinsic/autonomous motivation (Hagger and Chatzisarantis, 2011).

In addition, our results provide insights to existing literatures on autonomous motivation. While previous studies mainly examined effects of contextual factors and characteristics of the task itself on one’s autonomous motivation (Deci and Ryan, 1985b; Bergin, 1999; Deci and Ryan, 2000; Gagné, 2014), our findings suggested that actually there are interplays between prior experience and one’s motivation in the current activity. As it is a commonplace that a person would take part in several activities in a row, researchers may pay more attention to these dynamics and examine other influencing factors of one’s motivation from an intertemporal perspective (Meng and Ouyang, 2020; Newton et al., 2020; Wei et al., 2020).

Our findings bear important empirical implications as well. Previous literatures in the educational setting have consistently showed that students would display higher disengagement and more bullying behaviors when their teachers adopt a controlling instructional style (Hein et al., 2015; Jang et al., 2016; Earl et al., 2017). Similarly, autonomy frustration in the work domain would lead to employees’ emotional burnout, ill-being and counter-productive behavior (Van Den Broeck et al., 2014; Gillet et al., 2015a). In our study, we further found that autonomy frustration as a result of the controlling context would have a spillover effect on the subsequent activity. Thus, if the employer or instructor adopts a controlling style, then it is highly likely that the employees/students would display lower autonomous motivation not only in the current activity but also in future ones. While the negative impact of the controlling style can be profound and long-lasting, actually it is not uncommon in the practice. Given the current finding, it is imperative that managers/educators endeavor to cultivate and facilitate autonomy-supportive work/teaching environment instead. Besides, previous researches in the work domain suggested that, in addition to the controlling managerial style, work pressure and job uncertainty would also cause autonomy frustration (Gillet et al., 2015b). Thus, it is imperative for managers to try everything they can to protect their employees’ autonomy.

We would like to acknowledge some limitations of this study. First, given that the Tangram puzzle task was closely followed by the stopwatch task, the spillover effect of autonomy frustration on one’s motivation reported in this study was only short-term. In other words, the current experimental design did not allow us to examine the long-term effect of autonomy frustration. Second, as indicated in previous studies (Fang et al., 2018, 2019), people with different personalities (i.e., achievement goal orientation) or mindsets may vary in their cognitive and behavioral responses to psychological need frustration. While we found one’s control causality orientation to have an effect in the spillover effect of autonomy frustration in this study, other individual difference factors are worth investigating. Finally, while magnitudes of ERP components reflect one’s cognitive and emotional evaluation of experimental stimuli to a certain extent, neural representations of human motivation are quite complicated and dynamic. Thus, it is highly recommended that findings of this study are to be replicated using other methods (e.g., field experiment). These limitations suggest directions for future research.

## Conclusion

In a two-session experiment, we first manipulated experimental instructions to either introduce or not introduce autonomy frustration in the first task and then explored its effect on one’s autonomous motivation and attention level in a subsequent autonomy-supporting activity. Electrophysiological data suggested that following autonomy frustration, participants exhibited decreased autonomous motivation (as reflected by the amplitude of RewP difference wave) and reduced attention level (as measured by the magnitude of P300) in the subsequent autonomy-supportive activity. Negative correlation between one’s control causality orientation and the magnitude of RewP difference wave further illustrated that control orientation would aggravate the destructive spillover effect of autonomy frustration. Taken together, by examining the intertemporal effect of autonomy frustration, we extend existing literatures on the dynamics between autonomy frustration and one’s autonomous motivation and provide important practical implications.

## Data Availability Statement

The datasets generated for this study are available on request to the corresponding author.

## Ethics Statement

The studies involving human participants were reviewed and approved by the Internal Review Board of the Laboratory of Neuromanagement and Decision Neuroscience, Guangdong University of Technology. The participants provided their written informed consent to participate in this study.

## Author Contributions

LM and HF conceived and designed the study. HF collected and analyzed the data. HF and LM interpreted the data and drafted the manuscript. LM, HF, XW, and SZ reviewed and edited the manuscript. LM administered the project.

## Conflict of Interest

The authors declare that the research was conducted in the absence of any commercial or financial relationships that could be construed as a potential conflict of interest.
